# Synthesis of molecularly imprinted polymer by precipitation polymerization for the removal of ametryn

**DOI:** 10.1186/s13065-023-01084-0

**Published:** 2023-11-24

**Authors:** Rachel Marcella Roland, Showkat Ahmad Bhawani, Mohamad Nasir Mohamad Ibrahim

**Affiliations:** 1 Faculty of Resource Science and Technology, Universiti Malaysia Sarawak (UNIMAS), 94300 Kota Samarahan, Sarawak Malaysia; 2 School of Chemical Sciences, Universiti Sains Malaysia, 11800 Penang, Malaysia

**Keywords:** Ametryn, Herbicide, Molecular imprinted polymer, Precipitation polymerization, Removal, Tap water, River water

## Abstract

Ametryn (AME) is a triazine herbicide which is mainly used to kill unwanted herbs in crops. Despite its importance in agriculture, the usage of AME also poses a risk to humans and the ecosystem due to its toxicity. Hence, it is important to develop a method for the effective removal of AME from various water sources which is in the form of molecular imprinting polymer (MIP). In this study, MIP of AME was synthesized via precipitation polymerization using AME as the template molecule with three different functional monomers including methacrylic acid (MAA), acrylamide (AAm) and 2-vinylpyridine (2VP). The three different synthesized polymers namely MIP (MAA), MIP (AAm) and MIP (2VP) were characterized using Fourier Infra-red spectroscopy (FTIR) and Field Emission Electron Microscopy (FESEM). Then, the batch binding study was carried out using all three MIPs in which MIP (MAA) attained the highest rebinding efficiency (93.73%) among the synthesized polymers. The Energy-Dispersive X-ray spectroscopy (EDX) analysis, Brunauer–Emmett–Teller (BET) analysis and thermogravimetric analysis (TGA) were also conducted on the selected MIP (MAA). Adsorption studies including initial concentration, pH and polymer dosage were also conducted on MIP (MAA). In this study, the highest adsorption efficiency was attained at the optimum condition of 6 ppm of AME solution at pH 7 with 0.1 g of MIP (MAA). MIP (MAA) was successfully applied to remove AME from spiked distilled water, tap water and river water samples with removal efficiencies of 95.01%, 90.24% and 88.37%, respectively.

**Graphical Abstract:**

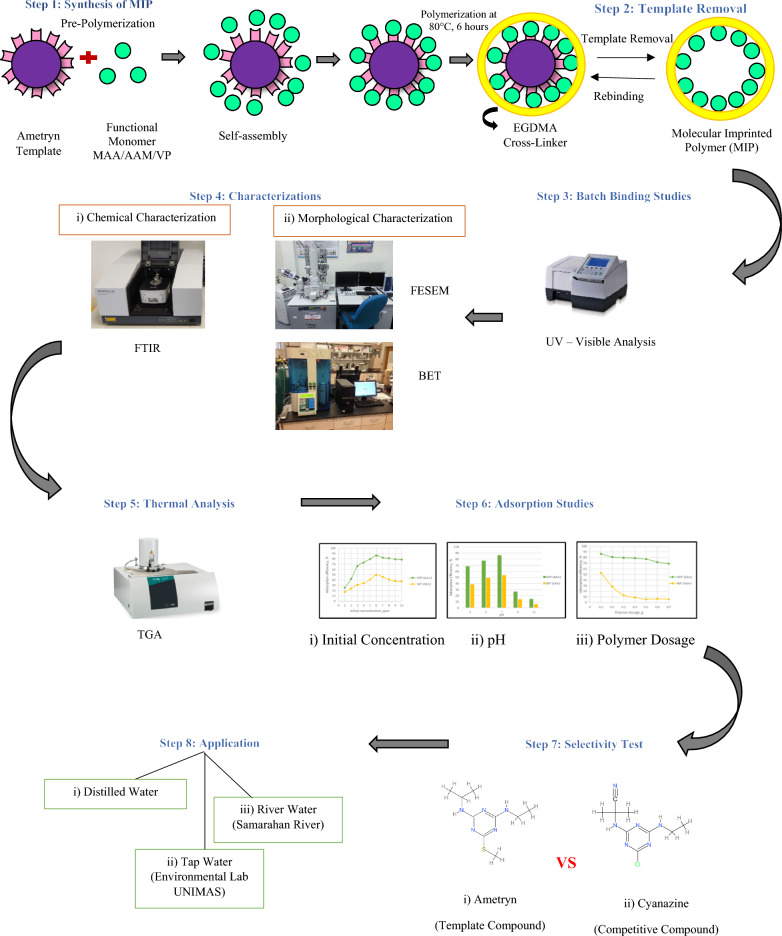

## Background

Herbicides also known as weedkillers are chemicals used to eliminate unwanted vegetation to increase the quality and yield of the crops. Herbicides are progressively utilized in agriculture and applied by other fields such as railway companies, landscapers, greenkeepers, sports field managers, municipalities, and private gardeners [1]. Herbicides are more persistent in the environment due to their bioaccumulation, lipophilicity, long half-life and numerous modes of transport for these chemicals [2].

Ametryn (AME) or chemically known as 4-*N*-ethyl-6-methylsulfanyl-2-*N*-propan-2-yl-1, 3,5-triazine-2,4-diamine is one of the methylthio-*s*-triazine herbicide [3] that was designed to inhibit photosynthesis of weeds in crops such as corn, pineapple and soybean [4, 5] as well as sugarcane [6]. This *s*-triazine herbicide is broadly used globally due to its efficiency in controlling the growth of broadleaf and grass weeds [7]. However, the U.S. Environmental Protection Agency (EPA) has classified AME as a Class III herbicide [8], indicating that it is slightly toxic due to its high solubility in water, weak adsorption behaviour, high environmental persistency (in soil and water), and very mobile in the environment [9]. For these reasons, AME is called an environmental contaminant as it can be detected in surface water [10–15], soil [7, 16] and groundwater [17]. Besides that, AME contamination in water compartments will be very challenging and eventually harmful to human health and the ecosystem such as animals, aquatic life, as well as water sources. For example, AME poisoning can cause nausea, diarrhoea, salivation, muscle weakness, dermatitis, eye irritation and respiratory tract irritation [5, 18]. In serious cases, prolonged exposure to AME can cause cancer due to its functionality as an endocrine-disrupting compound [18]. Moreover, the presence of AME in aquatic environments possesses high to moderate toxicity to fish, high toxicity to crustaceans and moderate to high toxicity to molluscs [19]. A lower abundance of various tadpole species was noticeably associated with the presence of AME in those agricultural areas [20]. In this regard, the removal of AME is essential to sustain a prolonged healthy and safe ecosystem for all living organisms and the environment.

Until recent years, many studies have been focussed on AME detection [6, 21–27], AME toxicity and its effects towards mankind [5, 18], amphibians [28–30], fish [31] and the environment [32, 33]. Numerous studies were also performed for AME degradation [2, 5, 7, 34–37], AME sorption [38] and desorption [39]. However, lack of studies conducted on the selective removal of AME from the environment which is very important for human health, animals, and environmental safety. For these reasons, the study of an effective method for the removal of contaminants from the environment is vital and this can be conducted by molecularly imprinted polymers (MIPs), a highly cross-linked synthetic polymer where the binding sites and cavities of the MIPs are corresponding to the template molecule or target compound [40]. MIPs are made up from co-polymerization between template molecule and functional monomer in the presence of excess cross-linker and initiator in a suitable solvent, followed by template removal from the polymer cavity to obtain the MIPs [41]. MIPs have been a versatile material for the effective removal of numerous contaminants such as melamine [42–44], organochlorine fungicides [45], 2-phenylphenol [46], 2,4,6-trichlorophenol [47], Congo red [48] and Sudan III [49]. Moreover, the green MIP principles also known as greenification [50–53] are essential for producing eco-friendly and sustainable methods for the production of MIPs which are then applied for environmentally responsible purposes, such as pollutant removal, water purification, or in green chemistry applications that can reduce the use of harmful chemicals.

Therefore, it is an ideal technique for AME removal from water samples because the MIP preparation is a straightforward and economical method which generates specific and selective polymers with high storage stability, mechanical strength, and durability in extreme chemical conditions [54]. The current challenge is preparation of water-compatible MIPs with high selectivity. This issue must be addressed in order to improve the efficiency and effectiveness of MIPs for the removal of herbicides from aqueous samples.

The main objective of this research was to synthesize molecularly imprinted polymers (MIPs) for the removal of AME from three water samples including distilled water, tap water and river water. In this study, toluene was selected as the porogenic solvent and three different functional monomers of different acidity/basicity were used, namely, methacrylic acid (MAA), acrylamide (AAm) and 2-vinylpyridine (2VP). MIPs were produced using a non-covalent approach via precipitation polymerization method. Generally, a non-covalent approach involves the non-covalent bond interactions between the AME template molecule and functional monomers such as MAA, AAm or 2VP. Van der Waals forces, hydrogen bonding, interactions, dipole–dipole interactions, and ion–dipole interactions are examples of non-covalent bonds that have weak interactions between the template molecule and functional monomer and thus require minimal synthetic effort for binding and template removal. Moreover, precipitation polymerization method was used in this work because it commonly produces micro-spherical polymer particles. This type of polymerization also generates a high yield of water-compatible polymers without the need for straining or crushing.

## Methods

### Materials

Ametryn (AME), cyanazine (CYZ), methacrylic acid (MAA), ethylene glycol dimethacrylate (EGDMA) and 2-vinyl pyridine (2VP) were purchased from Sigma-Aldrich. Acrylamide (Aam), azo-bis-isobutyronitrile (AIBN) and methanol (MeOH) were obtained from R & M Chemicals. Acetic acid (AcOH) and acetone (Ace) are purchased from Bendosen Laboratory Chemical. Acetonitrile (HPLC grade) was bought from Fisher Scientific. Distilled water (DIW) was used throughout the study.

### Synthesis of MIPs and NIP of AME

The polymers (MIPs and NIP) were synthesized by using the precipitation polymerization method using different types of functional monomers such as acidic, neutral, and basic with similar molar ratios was employed as given in Table 4.

A 100 mL of 1.0 mmol template solution (AME), 5.0 mmol of functional monomer (MAA), 20.0 mmol cross-linker (EGDMA) and 30 mg of initiator (AIBN) were poured into a 250 mL conical flask. The mixture was sonicated (Brandson 2510) for 10 min and then degassed with nitrogen in an ice water bath for 15 min. After that, the conical flask was sealed tightly and immersed in a water bath (Biobase XMTD-204) at 80 °C for 6 h to complete the polymerization reaction. The synthesized polymer particles were collected by centrifugation (Gyrozen-406) at 4000 rpm for 15 min and dried at room temperature, 25 °C. A similar experimental procedure was followed for the MIP preparation using different functional monomers as listed in Table 1. The non-imprinted polymer (NIP) was synthesized similarly but without the addition of the AME template molecule.

**Table 1:** Template–monomer–crosslinker ratios for synthesis of MIPs and NIP of AME

Polymer	Template	Monomer			Crosslinker	Initiator
		Acidic	Neutral	Basic		
	AME (mmol)	MAA (mmol)	AAm (mmol)	2VP (mmol)	EGDMA (mmol)	AIBN (mg)
MIP (MAA)	1	5	–	–	20	30
NIP (MAA)	–	5	–	–	20	30
MIP (AAm)	1	–	5	–	20	30
MIP (2VP)	1	–	–	5	20	30

### Removal of AME from synthesized MIPs

The synthesized MIPs were continuously washed with a mixture of MeOH and AcOH (6:4, v/v) until the AME template was not detected by UV–visible spectrophotometer (Agilent Cary 60). Then, the MIPs were rinsed with methanol to remove the acetic acid from the polymer matrix. Finally, the synthesized polymer particles were collected by centrifugation at 4000 rpm for 15 min and dried at room temperature, 25 °C. Only MIPs were washed with a mixture of MeOH and AcOH to remove the AME template. The NIP was not required to wash with a mixture of MeOH and AcOH to remove the AME template since no AME template was added during the NIP synthesis.

### Characterization of MIPs and NIP of AME

FT-IR analysis (Thermo Scientific Nicolet iS10 FTIR with Diamond ATR) was conducted on both MIPs and NIP of AME to identify and compare the functional groups present in the IR spectral range of 4000–500 cm^−1^.

FESEM and EDX analyses (JOEL JSM-IT500HR) were carried out to observe the morphology (size and shape) and detect the composition of elements present in the synthesized polymers at a magnification of 10k, respectively.

BET analysis (Brunauer–Emmett–Teller Quantachrome Autosorb) was also performed on both MIPs and NIP to measure the specific surface area, pore volume and average pore diameter of the synthesized polymers.

Thermal analysis (Universal Analyzer 2000 with Universal V4.7A software) was employed at a temperature range of 30–900 °C at the heating rate of 10 °C/min to determine the stability temperature of the imprinted polymer.

### Batch binding of MIPs and NIPs of AME

A batch binding study was carried out to determine the highest rebinding efficiency of AME with different MIPs and NIP. Initially, a 5 ppm AME solution was prepared by dissolving AME template in a mixture of MeOH and DIW (1:9, v/v). A series of 100 mL conical flasks containing 0.1 g of MIPs and NIP were added with 10 mL of 5 ppm AME solution, respectively. The conical flasks containing the mixture were agitated on an orbital shaker (Heathrow Scientific) at the speed of 150 rpm and the samples were collected at different time intervals (0, 5, 10, 15, 20, 25, 20, 60, 90, 120, 150, 180, 210, 240, 270 and 300 min). The concentrations of AME after the rebinding were observed by using UV–visible spectrophotometer.

The rebinding efficiencies of MIPs and NIP of AME were evaluated by using the following Eq. (1):

1 $${\text{Binding}}\;{\text{efficiency}} = \left[ {\left( {{\text{C}}_{{\text{i}}} - {\text{C}}_{{\text{f}}} } \right)/{\text{C}}_{{\text{i}}} } \right] \times 100\% ,$$

where C_i_: the initial AME concentration in the solution before binding, and C_f_: the final AME concentration in the solution after binding.

### Adsorption studies MIP and NIP of AME

For adsorption studies (initial concentration, pH, and polymer dosage), only MIP (MAA) and its NIP (MAA) were selected based on the batch binding study because MIP (MAA) had the highest rebinding efficiency among the imprinted polymers such as MIP (AAm) and MIP (2VP).

### Effect of initial concentration

Firstly, AME solutions of different concentrations (1, 2, 3, 4, 5, 6, 7, 8, 9 and 10 ppm) were prepared by dissolving AME template in a mixture of MeOH and DIW (1:9, v/v). Then, a 0.1 g of MIP and NIP beads were added into a series of 100 mL conical flasks containing 10 mL of different AME concentrations. The conical flasks containing the mixture were agitated on an orbital shaker at the speed of 150 rpm for 210 min. The concentrations of AME after the rebinding were observed by using UV–visible spectrophotometer. The rebinding efficiencies of MIP and NIP of AME were evaluated by using the following Eq. (1).

### Effect of pH

A 0.1 g of MIP and NIP beads were added into a series of 100 mL conical flasks containing 10 mL of 7 ppm of AME solution (MeOH: DIW, 1:9, v/v) at different pH (pH 3, pH 5, pH 7, pH 9 and pH 11). The pH of AME solution was adjusted by using 5% hydrochloric acid to attain acidic condition while 5% sodium hydroxide was used to attain basic condition. The conical flasks containing the mixture were agitated on an orbital shaker at the speed of 150 rpm for 210 min. The concentrations of AME after the rebinding were observed by using UV–visible spectrophotometer. The rebinding efficiencies of MIP and NIP of AME were evaluated by using the following Eq. (1).

### Effect of polymer dosage

Different amounts of MIP and NIP beads (0.1 g, 0.2 g, 0.3 g, 0.4 g, 0.5 g, 0.6 g and 0.7 g) were added into a series of 100 mL conical flasks containing 10 mL of 7 ppm of AME solution (MeOH: DIW, 1:9, v/v) at pH 7. The conical flasks containing the mixture were agitated on an orbital shaker at the speed of 150 rpm for 210 min. The concentrations of AME after the rebinding were observed by using UV–visible spectrophotometer. The rebinding efficiencies of MIP and NIP of AME were evaluated by using the following Eq. (1).

### Kinetic studies of MIP of AME

Three different kinetic models such as pseudo-first-order, pseudo-second-order and intraparticle-diffusion were applied in this study to investigate the rate and kinetic mechanism of MIP (MAA) adsorption. Equations (2), (3) and (4) represented the linear equations of pseudo-first-order, pseudo-second-order and intraparticle-diffusion, respectively. Among these kinetic models, only one kinetic model with a higher correlation coefficient (R^2^) was selected as the best-fitted kinetic model which describes the adsorption of MIP (MAA).

2 $${\mathbf{Pseudo}\text{-}\mathbf{first}\text{-}\mathbf{order}}:\log \left( {{\text{q}}_{{\text{e}}} {-}{\text{q}}_{t} } \right) = \log \left( {{\text{q}}_{{\text{e}}} } \right){-}{\text{K}}_{1} \left( {t/2.303} \right),$$

where q_e_ = the amount of AME adsorbed at equilibrium time, q_*t*_ = the amount of AME adsorbed at any given time *t*, K_1_ = the pseudo-first-order equilibrium rate constant, and *t* = the time interval of AME adsorption.

3 $${\mathbf{Pseudo}\text{-}\mathbf{second}\text{-}\mathbf{order}}:{\text{t}}/{\text{q}}_{t} = 1/{\text{K}}_{2} \left( {{\text{q}}_{{\text{e}}} } \right)^{2} + {\text{t}}/{\text{q}}_{{\text{e}}} ,$$

where q_e_ = the amount of AME adsorbed at equilibrium time, q_*t*_ = the amount of AME adsorbed at any given time *t*, K_2_ = the pseudo-second-order equilibrium rate constant, and *t* = the time interval of AME adsorption.

4 $${\mathbf{Intraparticle}\text{-}\mathbf{diffusion}}:{\text{q}}_{t} = {\text{K}}_{{{\text{dif}}}} \surd t + C,$$

where q_*t*_ = the amount of AME adsorbed at any given time *t*, K_dif_ = the intraparticle equilibrium rate constant, *t* = the time interval of AME adsorption, and *C* = another kinetic constant.

### Isotherm studies of MIP of AME

Two isotherm models such as Langmuir and Freundlich isotherm models were employed in this study to describe the adsorption capability and surface of the MIP (MAA). Equations (5) and (7) represented the linear equations of Langmuir and Freundlich isotherm models, respectively. Between these two isotherm models, only one model with a higher correlation coefficient (R^2^) was selected as the best-fitted isotherm model.

5 $${\mathbf{Langmuir}}\;{\mathbf{Isotherm}}:{\text{C}}_{{\text{e}}} /{\text{q}}_{e} = \left( {1/{\text{q}}_{\max } {\text{K}}_{{\text{L}}} } \right) + \left( {{\text{C}}_{{\text{e}}} /{\text{q}}_{\max } } \right),$$

where C_e_ = the AME concentration at equilibrium, q_e_ = the amount of AME adsorbed at equilibrium time, q_max_ = the maximum adsorption capacity of MIP (MAA) and K_L_ = the Langmuir constant.

Moreover, the R_L_ value (parameter of equilibrium) related to the fundamental characteristics of the Langmuir isotherm model was calculated by using Eq. (6)

6 $${\text{R}}_{{\text{L}}} = 1/\left( {1 + {\text{K}}_{{\text{L}}} {\text{C}}_{{\text{e}}} } \right),$$

where K_L_ = the Langmuir constant and C_e_ = the AME concentration at equilibrium.

7 $${\mathbf{Freundlich}}\;{\mathbf{isotherm}}:{\text{In}}\left( {{\text{q}}_{e} } \right) = {\text{In}}\left( {{\text{K}}_{{\text{F}}} } \right) + \left( {1/n} \right)\left( {{\text{InC}}_{{\text{e}}} } \right),$$

where C_e_ = the AME concentration at equilibrium, q_e_ = the amount of AME adsorbed at equilibrium time, K_F_ = the Freundlich constant and *n* = constants associated with the intensity of adsorption.

### Selectivity test of MIP of AME

The two conical flasks were taken in which one contained 0.1 g of MIP (MAA) and another with 0.1 g of NIP (MAA). Then a 10 mL mixture of 7 ppm AME (MeOH: DIW, 1:9, v/v) and 7 ppm CYZ solution (MeOH: DIW, 1:9, v/v) added in both the flasks. Then, the conical flasks were agitated on an orbital shaker at 150 rpm for 210 min. The concentrations of AME after the rebinding were observed by using UV–visible spectrophotometer. The selectivity of MIP and NIP towards AME were expressed in terms of the distribution ratio (K_D_), using the following Eq. (8).

8 $${\text{K}}_{{\text{D}}} = \left[ {\left( {{\text{C}}_{{\text{i}}} {-}{\text{C}}_{{\text{f}}} } \right)/{\text{C}}_{{\text{f}}} } \right]\left( {{\text{V}}/{\text{m}}} \right),$$

where C_i_: the initial AME concentration in the solution before adsorption, C_f_: the final AME concentration in the solution after adsorption, V: the volume of solvent used, and m: the mass of MIP/NIP used.

The selectivity coefficients for AME (template molecule) relative to CYZ (binding competitor) for MIP and NIP were determined according to Eq. (9).

9 $${\text{Selectivity}}\;{\text{coefficient}},{\text{ K}}_{{{\text{sel}}}} = {{{\text{K}}_{{\text{D}}}^{{{\text{AME}}}} } \mathord{\left/ {\vphantom {{{\text{K}}_{{\text{D}}}^{{{\text{AME}}}} } {{\text{K}}_{{\text{D}}}^{{{\text{CYZ}}}} }}} \right. \kern-0pt} {{\text{K}}_{{\text{D}}}^{{{\text{CYZ}}}} }},$$

where ${\text{K}}_{{\text{D}}}^{{{\text{AME}}}}$: the batch binding assay of MIP/NIP for AME, and ${\text{K}}_{{\text{D}}}^{{{\text{CYZ}}}}$: the batch binding assay of MIP/NIP for CYZ.

Hence, the relative selectivity coefficient (K′) was defined by Eq. (10), as below.

10 $${\text{K}}^{\prime} = {\text{KMIP}}/{\text{KNIP}}.$$

### Removal of AME from tap water and river water

The collected tap water (Environmental Laboratory 1, Faculty of Resource Science and Technology) and river water (Samarahan River) were filtered by using vacuum filtration to eliminate any suspended particles. Any presence of AME in the collected tap water and river water was monitored using a UV–visible spectrophotometer.

Firstly, the filtered river water, tap water and DIW were spiked with 25 µg/mL of AME, respectively. Secondly, 0.1 g of MIP (MAA) and NIP (MAA) were added to respective conical flasks containing 10 mL of spiked AME solution. The conical flasks containing the mixture were agitated on a shaker at 150 rpm for 210 min. Finally, the AME concentrations after adsorption were observed via UV–visible spectrophotometer. The AME removal efficiency in river water, tap water and DIW by using MIP (MAA) and NIP (MAA) were calculated using Eq. (1).

## Results and discussions

### FT-IR analysis

**Fig. 1:**
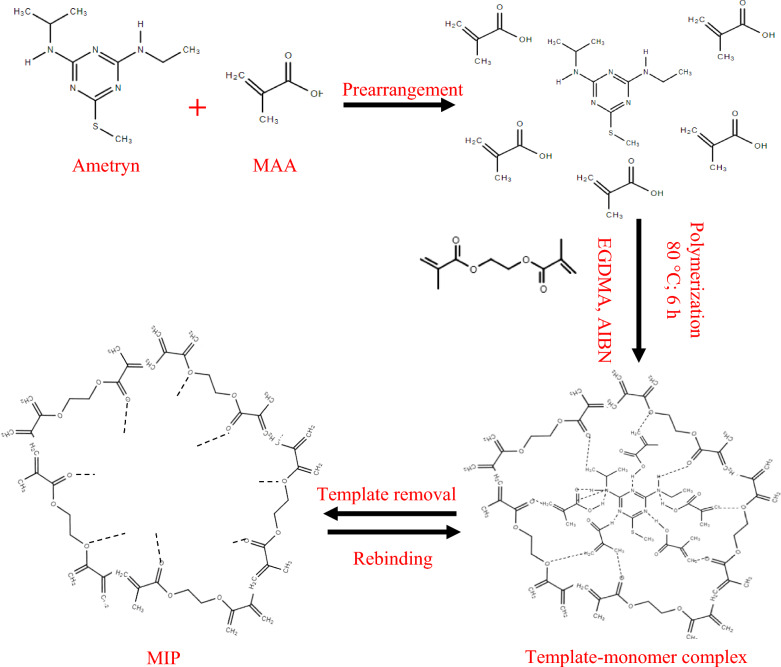
Proposed mechanism of polymerization reaction

**Fig. 2:**
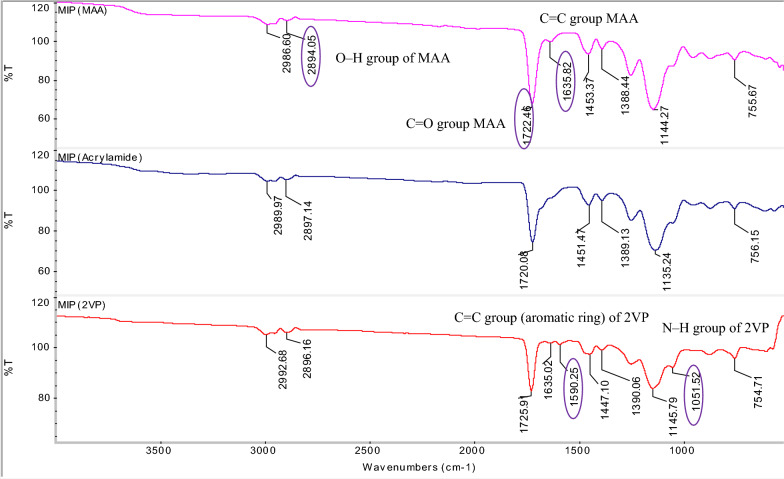
FTIR spectra of MIPs of AME

**Fig. 3:**
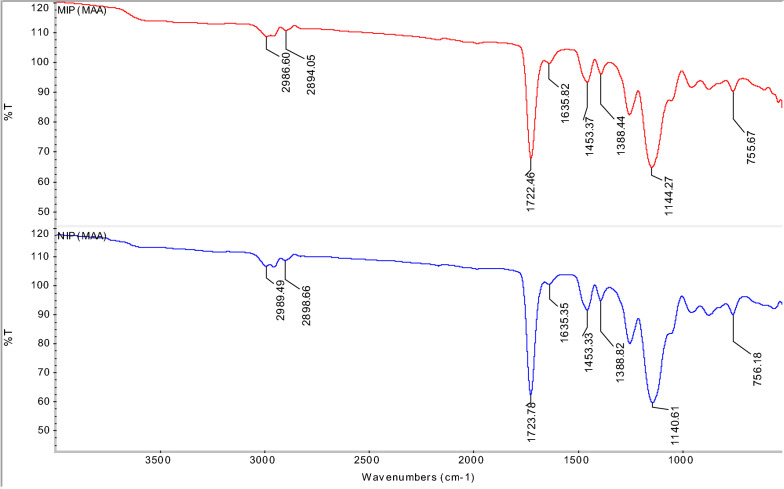
FTIR spectra of MIP (MAA) and NIP (MAA) of AME

In MIP development, FTIR is employed to distinguish the chemical bonds formed (particularly Hydrogen bonding) among the template molecule and functional monomer based on the IR spectra of the polymer sample and peak shifting [55, 56]. The FTIR analysis on MIPs and NIP indicated the functional groups present in the synthesized polymers due to the interaction among template molecule (AME), functional monomer (MAA/AAm/2VP) and cross-linker (EGDMA). Figure 1 shows the general mechanism of MIP synthesis involving binding interactions among AME as the template molecule, MAA as one of the functional monomers, and EGDMA as the cross-linker to produce MIP with specific binding sites. Hence, the peaks attributed to these chemical compounds were anticipated to be available in the IR spectra as shown in Figs. 2 and 3.

The medium peak at 2986.60 cm^−1^, 2994.09 cm^−1^ and 2992.68 cm^−1^ may indicate the C–H stretching of the alkyl group of AME (template molecule) and EGDMA (cross-linker) in IR spectra of MIP (MAA), MIP (AAm) and MIP (2VP), respectively. Besides that, the peak at 2986.60 cm^−1^ of MIP (MAA) might also imply the O–H stretching of the carboxylic acid group in methacrylic acid (MAA), a functional monomer. Similarly, the small peaks at 2894.05 cm^−1^, 2897.14 cm^−1^ and 2896.16 cm^−1^ may refer to the C–H stretching of the alkyl group of AME and EGDMA in MIP (MAA), MIP (AAm) and MIP (2VP), respectively. The peak of MIP (MAA) at 2897.14 cm^−1^ might also denote the O–H stretching of the carboxylic acid group in MAA. These peaks may be assigned to several functional groups including the alkyl functional group [57] in the AME template molecule and EGDMA cross-linker, as well as the hydroxyl functional group that is present in the MAA cross-linker. This proved the incorporation among AME template molecule and EGDMA cross-linker for MIP (AAm) and MIP (2VP), as well as interaction of MAA functional monomer in MIP (MAA).

A strong peak appeared in IR spectra of MIP (MAA), MIP (AAm) and MIP (2VP) at 1722.48 cm^−1^, 1720.08 cm^−1^ and 1725.91 cm^−1^, respectively, and these peaks may represent the C=O stretching [58, 59] of ester group in EGDMA, cross-linker. Besides that, the peak of MIP (MAA) at 1722.48 cm^−1^ may show the presence of C=O stretching of the carboxylic acid group in MAA. The weak peak of MIP (MAA) at 1635.82 cm^−1^ and MIP (2VP) at 1635.02 cm^−1^ may display the presence of C=C stretching of the alkenyl group [60] in MAA and 2VP, respectively and no peak of MIP (AAm) was detected at this adsorption band.

Only MIP (2VP) had a weak peak at 1590.25 cm^−1^ and this assigned to C=C stretching of the aromatic ring [60] in 2-vinylpryridine. A medium peak in IR spectra of MIP (MAA), MIP (AAm) and MIP (2VP) at 1453.37 cm^−1^, 1451.47 cm^−1^ and 1447.10 cm^−1^, respectively, may prove the C=C stretching of the aromatic ring [60] in AME template. These peaks might also be attributed to the –CH_2_ bending and –CH_3_ bending of MAA, AAm and 2VP in MIP (MAA), MIP (AAm) and NIP (AAm), respectively as well as the EGDMA. The same peak at 1447.10 cm^−1^ of MIP (2VP) might also indicate the presence of C=C stretching of the aromatic ring [61] in 2-vinylpryridine.

Another medium peak at 1388.44 cm^−1^, 1389.13 cm^−1^ and 1390.66 cm^−1^ may indicate the –CH_3_ bending of the alkane group in the AME template and EDGMA cross-linker as shown in IR spectra of MIP (MAA), MIP (AAm) and MIP (2VP). The peak of MIP (MAA) at 1388.44 cm^−1^ may also show the CH_3_ bending of the alkane group in MAA, a functional monomer. Moreover, this peak at 1388.44 cm^−1^ might imply the presence of –CH_3_ bending of the alkane group in MAA, the functional monomer of MIP (MAA). A strong peak at 1144.27 cm^−1^, 1135.24 cm^−1^ and 1447.10 cm^−1^ of MIP (MAA), MIP (AAm) and MIP (2VP), respectively, may represent the C–O–C stretching of ester in EGDMA and also the C–N stretching of amine group in AME template. Only IR spectra of MIP (2VP) had a peak at 1051.52 cm^−1^, showing the presence of C–N stretching [61] of the amine group in 2VP, functional monomer. The peaks of MIP (MAA), MIP (AAm) and MIP (2VP) at 755.67 cm^−1^, 756.15 cm^−1^ and 754.71 cm^−1^ may show the C–H bending of the alkene group in EGDMA.

Figure 3 shows the comparison between the IR spectra of MIP (MAA) and NIP (MAA). Among the three synthesized MIPs, MIP (MAA) had the highest binding efficiency towards AME, therefore its NIP (MAA) was synthesized and the comparison of IR spectra between the MIP (MAA) and its NIP (MAA) were evaluated. Since MIP (MAA) was synthesized in the presence of an AME template molecule, it was expected that a slight absorption band shifting might occur in comparison with the NIP (MAA) that was synthesized without the presence of an AME template molecule.

In IR spectra of MIP (MAA), a medium peak at 2894. 05 cm^−1^ and 2986.60 cm^−1^ may assign to the C–H stretching of the alkyl group [57] of AME (template molecule) and EGDMA (cross-linker) as well as the O–H stretching of the carboxylic acid group in methacrylic acid (MAA), functional monomer. While in IR spectra of NIP (MAA), a slight shifting of the peak at 2898.66 cm^−1^ and 2989.49 cm^−1^ indicates the C–H stretching of the alkyl group [57] of EGDMA and the O–H stretching of the carboxylic acid group in MAA.

A strong peak at 1722.46 cm^−1^ in MIP (MAA) shows the C=O stretching of the ester group [58, 59] and the carboxylic acid group in EGDMA and MAA, respectively. A small peak shifting at 1723.78 cm^−1^ in NIP (MAA) displays the presence of C=O stretching of the ester group and C=C stretching of the alkenyl group in EGDMA and MAA, respectively. The peaks present from 1700 to 1750 cm^−1^ showed the C=O stretching of the crosslinking polymerization between the EGDMA crosslinker and MAA monomer [62]. A weak peak of MIP (MAA) at 1635.82 cm^−1^ implies the presence of the C=C stretching of the alkenyl group in MAA. A minor peak shifting at 1635.82 cm^−1^ of NIP (MAA) was observed indicating the C=C stretching of the alkenyl group in MAA. The peak of MIP (MAA) at 1453.37 cm^−1^ represents the C=C stretching of the aromatic ring in the AME template as well as the –CH_2_ bending and –CH_3_ bending of MAA and EGDMA. Due to the absence of the AME template in the NIP (MAA), a very small shifting of the peak at 1453.33 cm^−1^, indicates the –CH_2_ bending and –CH_3_ bending of MAA and EGDMA.

In addition, the MIP (MAA) peak at 1388.44 cm^−1^ may display the –CH_3_ bending of the alkane group in the AME template, MAA and EDGMA. A small shift in NIP (MAA) at 1388.82 cm^−1^, might show the –CH_3_ bending of the alkane group in MAA and EDGMA only. The IR spectra of MIP (MAA) have a strong peak at 1144.27 cm^−1^ that is assigned to the C–O–C stretching of ester and C–N stretching of amine group in EGDMA and AME template, respectively. However, a strong peak of NIP (MAA) was shifted at 1140.61 cm^−1^ due to the C–O–C stretching of ester in EGDMA and no presence of AME template. The small peaks of MIP (MAA) and NIP (MAA) at 755.67 cm^−1^ and 756.18 cm^−1^, respectively, may imply the C–H bending of the alkene group in EGDMA.

### FESEM analysis

**Fig. 4:**
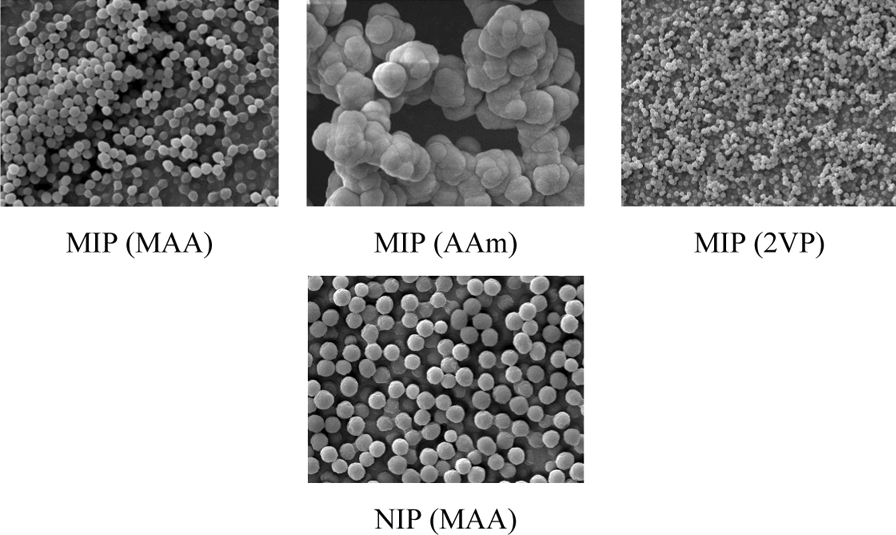
FESEM micrographs of synthesized MIPs and NIP at ×10,000 magnification

The FESEM analysis on MIPs and NIP showed the surface morphologies of synthesized MIPs and NIP at a magnification of 10k as shown in Fig. 4. Based on Fig. 4, the synthesized MIP (MAA), MIP (2VP) and NIP (MAA) were regular in size and spherical in shape. This is due to the precipitation polymerization method which synthesizes polymers in a circular shape and particle size within a particular range [62]. However, the synthesized MIP (AAm) was not only irregular in shape and size but also the polymer particles were agglomerated even though the precipitation polymerization method was used. This may be due to the high concentration of co-monomers (the total concentration of functional monomer and the cross-linker) that constrain the particle growth [63].

It was calculated (Table 2) that the mean diameter sizes of MIP (MAA), MIP (AAm) and MIP (2VP) were 0.52 µm, 1.23 µm and 0.36 µm, respectively. The mean diameter sizes of the synthesized MIPs were arranged in ascending order: MIP (2VP) < MIP (MAA) < MIP (AAm). This showed that MIP (2VP) had the smallest polymer particle size, followed by the MIP (MAA), and MIP (AAm) had the largest polymer particle size among the imprinted polymers.

**Table 2:** The FESEM analysis of synthesized MIPs and NIP

Polymer	Diameter, µm	Mean diameter, µm
MIP (MAA)	0.54	0.52
	0.49	
	0.52	
NIP (MAA)	0.82	0.80
	0.79	
	0.80	
MIP (AAm)	1.26	1.23
	1.32	
	1.11	
MIP (4VP)	0.32	0.36
	0.37	
	0.38	

Since MIP (MAA) attained its highest binding efficiency towards AME as compared to MIP (AAm) and MIP (2VP), the NIP (MAA) was analysed using FESEM to study the influence of imprinting effect of AME in the synthesized MIP (MAA). The mean diameter size of MIP (MAA) was 0.52 µm, which was smaller than that of NIP (MAA) of 0.80 µm as shown in Table 2. The presence of template molecules in the MIP polymerization reaction influenced the particle size of the synthesized MIP [64]. In addition, smaller particle sizes of polymers had a larger surface area—to volume ratio and therefore it had better binding capability with more substance on its surface [65].

### EDX analysis

**Fig. 5:**
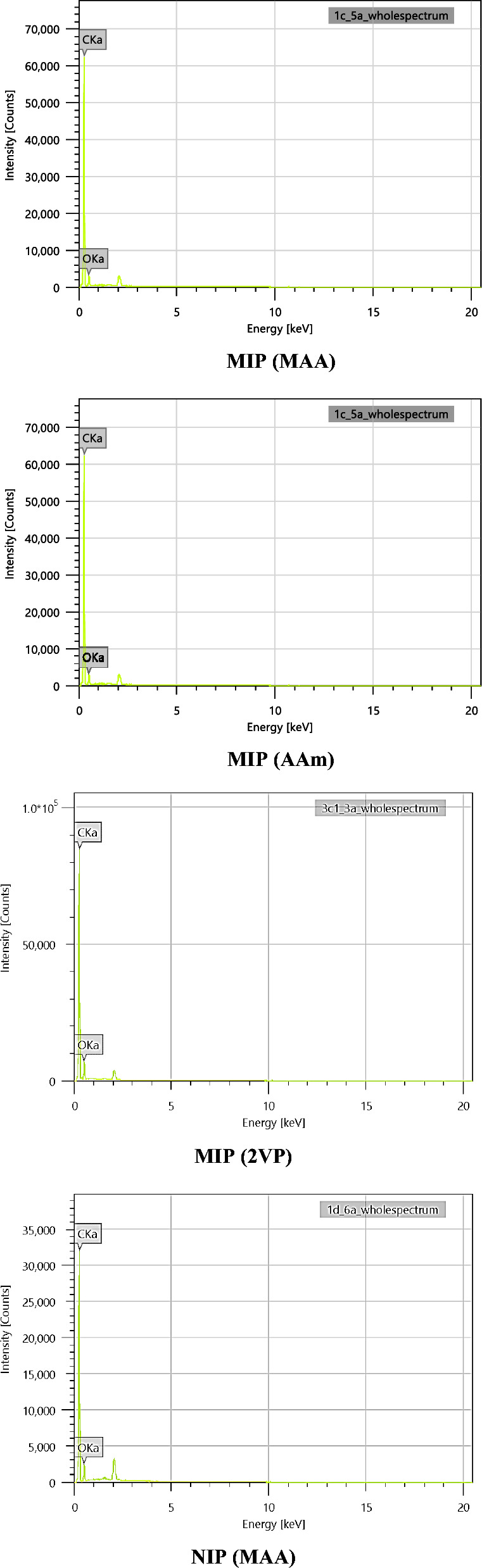
EDX analysis of synthesized MIPs and NIP

EDX analysis is another important study to determine the elemental compositions in the synthesized polymers [66]. The EDX analysis of MIP (MAA) demonstrated the mass percentage of the main chemical element namely carbon (C) and oxygen (O). Based on Fig. 5, the mass percentage of the C element in MIP (MAA) was 88.52%, much higher than that O element which was only 11.48%. The mass percentage of C and O elements in MIP (AAm) were 81.58% and 18.42%, respectively as shown in Fig. 5. Besides that, the mass percentage of C and O elements in MIP (2VP) were 82.60% and 17.40%, respectively (Fig. 5). Based on the EDX analysis of NIP (MAA) in Fig. 5, the C content was 83.51% while the O content was only 16.49%. These results indicated that the MIPs and NIP were made up of C as the main element in the polymer backbone.

### BET analysis

The BET analysis displayed the morphological characterizations of MIP (MAA) and NIP (MAA) in terms of surface area, average pore radius and total pore volume. Prior to this analysis, FESEM was performed to provide the morphology such as shape and size of synthesised polymers. The BET analysis is the extension of physical characterization of the synthesized MIP which provides a deeper understanding of the surface area and porosity which can influence the binding performance of polymers. Overall, the surface area, average pore radius and total pore volume of MIP (MAA) were larger than that of NIP (MAA) as shown in Table 3. This is due to the presence of a template molecule that generated several specific interactions during the polymerization process of the MIP [67]. A larger surface area of MIP than that of NIP showed that the specific binding sites were generated in the MIP cavities for the recognition of the template compound, resulting in greater MIP adsorption capacity [68].

**Table 3:** The BET analysis of MIP (MAA) and NIP (MAA)

Properties	Magnitude MIP (MAA)	Magnitude NIP (MAA)
Surface area (m^2^/g)	13.6801	11.593
Average pore radius (Å)	103.1369	92.9228
Total pore volume (cm^3^/g)	0.03178	0.029892

### TGA analysis

**Fig. 6:**
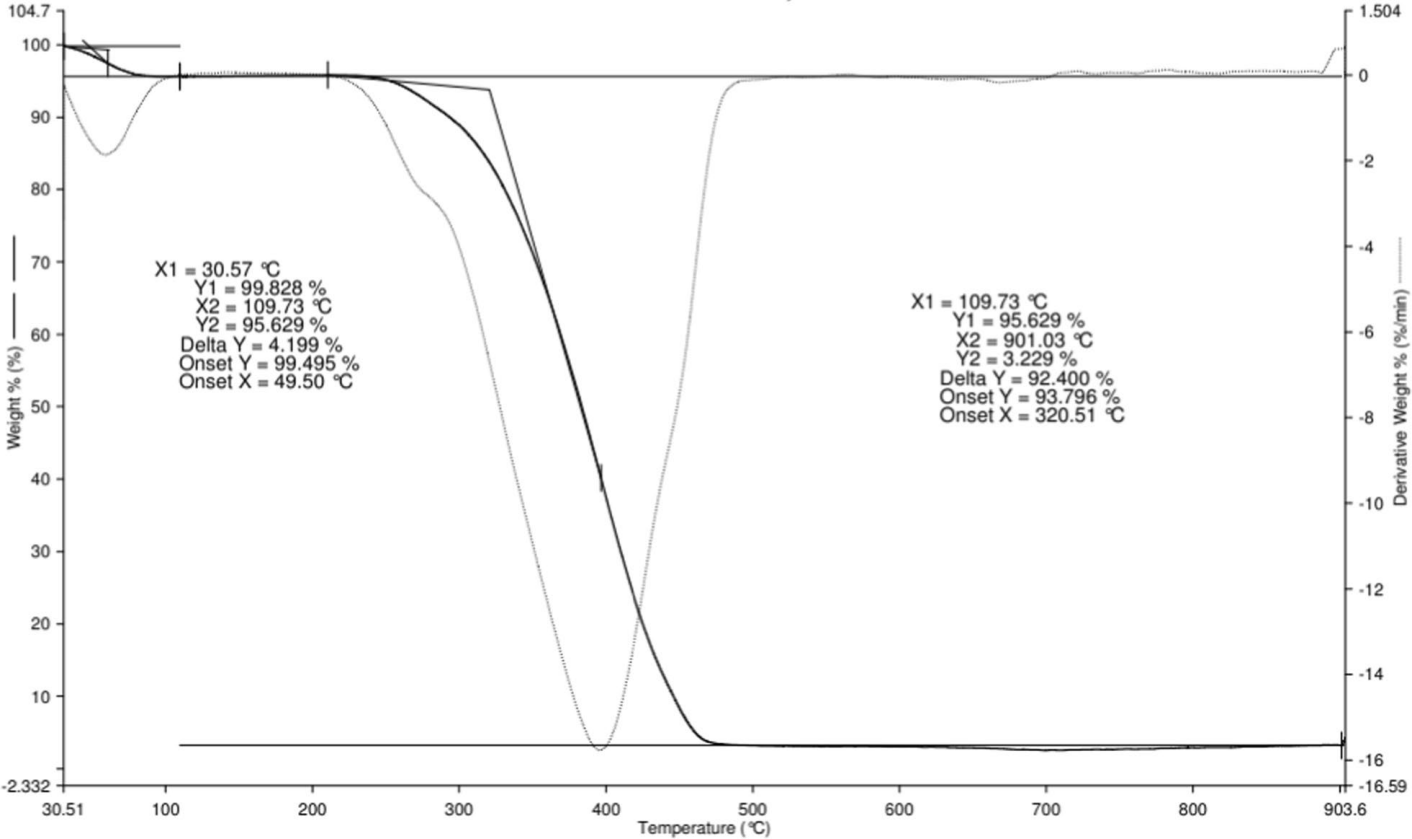
The TGA analysis of MIP (MAA) of AME

The thermogravimetric analysis (TGA) provides the study of the decomposition characteristics of the synthesized polymers [69]. The thermal degradation activity MIP (MAA) was performed from ~ 30 to ~ 900 °C, in an atmosphere of nitrogen as shown in Fig. 6. Based on Fig. 6, the MIP (MAA) had a slight weight loss of about 4% between 30.57 and 109.73 °C which is mainly due to the moisture or water. This indicated the first stage of weight loss in the MIP (MAA). Then, the weight of MIP (MAA) remained constant from 109.73 to 320.51 °C. However, a drastic weight loss of ~ 92% was observed from 320.51 to ~ 480 °C which was due to the degradation of the main polymer framework. This showed that the MIP (MAA) underwent the second weight loss. After that, the curve of MIP (MAA) remained consistent until ~ 900 °C, attributed to its thermal resistance whereby the amount of MIP (MAA) left was about 3%.

### Batch binding analysis

**Fig. 7:**
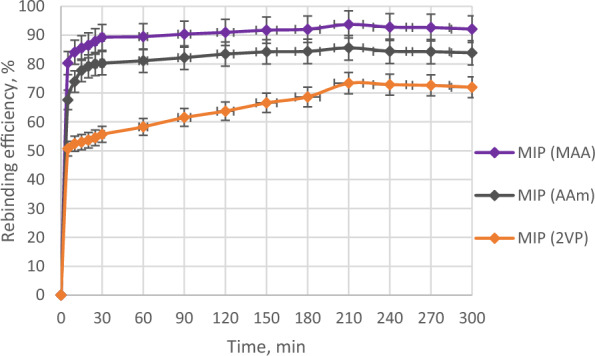
The batch binding analysis of all MIPs of AME

The batch binding analysis exhibited the rebinding efficiencies of the three different MIPs with AME at various contact times as shown in Fig. 7. Based on Fig. 7, the amount of AME adsorbed by the three MIPs increased with the rebinding time, up to a maximum of 210 min and after that, the adsorption of AME steadily decreased until 300 min. Numerous vacant binding sites and less mass transfer resistance in the polymer cavity permit the active sites to capture the target analyte (template molecule), increasing the rate of adsorption of the analyte on the surface of synthesized polymers, and hence increasing the binding efficiency [70]. Nevertheless, the number of active sites in the polymer cavity reduced with the increase of contact time as the binding sites were progressively attached to the analyte (template molecule) and the mass transfer resistance of the analyte to the binding sites also increased, causing the decrease in the rate of adsorption, and thus a slow decrease in binding efficiency [71].

As the contact time increased, more specific binding sites in the MIPs matrix could be occupied by the AME template molecule until it reached its saturation, resulting in the highest rebinding efficiencies at 210 min. The increase in contact time of the binding process leads to an increase in the adsorption of the target analyte onto the synthesized polymeric surface until the equilibrium is attained [72]. However, after this point of saturation (210 min), a further increase in contact time led to no rebinding of the template or a slight decrease in the polymer rebinding efficiencies because the available binding sites in the polymer matrix had been completely occupied with AME. Since the binding sites in the polymeric surface have been adequately occupied with the target analyte, the extended contact time for the binding process did not affect its percentage efficiency [70].

At 210 min, MIP (MAA), MIP (AAm) and MIP (2VP) attained their highest rebinding efficiencies with AME which were 93.73%, 85.61% and 73.87%, respectively as shown in Fig. 7. This indicated that the best contact time for the rebinding of MIPs with AME template was 210 min. When the adsorption phase of an analyte (template molecule) by the imprinted polymers had achieved its equilibrium, no obvious changes were observed in equilibrium concentration with an additional rise in contact time [43].

Among these three MIPs, MIP (MAA) had the highest rebinding efficiency with AME while MIP (2VP) had the lowest rebinding efficiency with AME. A suitable choice of functional monomer used in MIP synthesis allows stronger interactions between the template molecule and functional monomer which could generate a more stable template–monomer complex before the polymerization takes place, hence, resulting in the production of MIPs with greater imprinting efficiency [72–74]. So, this showed that MIP made up of AME (template molecule) with MAA (acidic functional monomer) was the best combination of template–monomer to obtain the highest rebinding efficiency.

**Fig. 8:**
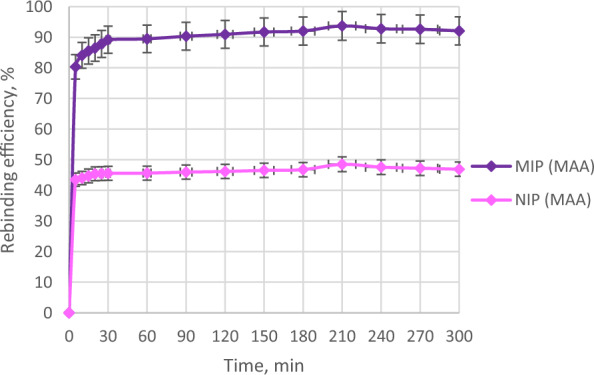
The batch binding analysis of MIP (MAA) and NIP (MAA) of AME

Figure 8 displays the comparison of the rebinding efficiencies between MIP (MAA) and NIP (MAA). Both MIP (MAA) and NIP (MAA) depicted a similar trend as the rebinding efficiencies of imprinted and non-imprinted polymers were increased from 0 to 210 min and then further decreased until 300 min.

The highest rebinding efficiencies of both MIP (MAA) and NIP (MAA) were 93.73% and 48.52%, respectively. MIP (MAA) had a much higher rebinding efficiency than NIP (MAA) due to the imprinting effect of AME in the MIP (MAA) cavity. The MIP particles with a larger surface area and total pore volume displayed a good imprinting effect [75]. The imprinting effect is due to the addition of a template molecule during MIP synthesis which produces more specific binding sites complementary to the template molecule [44].

As expected, NIP (MAA) had much lower rebinding efficiency than MIP (MAA) due to no imprinting effect as AME was absent during NIP synthesis, hence the lack of specific binding sites in the NIP cavity for the rebinding with AME.

### Adsorption studies

The adsorption studies of MIP (MAA) were divided into three parameters namely initial concentration, pH, and polymer dosage.

### Effect of initial concentration

**Fig. 9:**
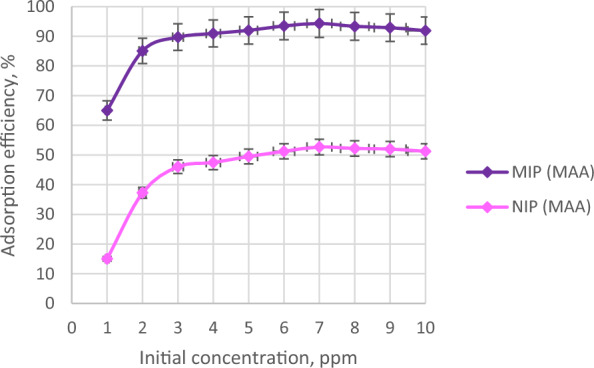
The adsorption efficiencies of MIP (MAA) and NIP (MAA) at different initial concentrations

Different initial concentrations of AME were used to study its effect towards the adsorption efficiency of the synthesized polymers. Figure 9 describes the adsorption efficiency of MIP (MAA) and NIP (MAA) at different AME initial concentrations (1 ppm to 10 ppm). It revealed that as the AME initial concentration increased, the adsorption efficiencies also increased, however, the adsorption efficiency had slightly decreased at a much higher AME initial concentration.

Based on Fig. 9, the adsorption efficiencies of MIP (MAA) towards AME were increased regularly from 1 to 7 ppm, then decreased gradually until 10 ppm. As the initial concentration of AME increased, the possibility for greater adsorption by MIP (MAA) also increased until it reached its saturation (at 7 ppm). The increment of AME adsorption efficiency by MIP (MAA) starting from 1 to 7 ppm indicated that there were plenty of binding sites present in the MIP (MAA) cavity that can bind with AME template molecule. Another study also mentioned that the MIP binding sites can be easily attached to the template molecule in increasing analyte (template molecule) concentration, and hence, promote better adsorption of the template molecule by the synthesized MIP [76].

The highest adsorption efficiencies of MIP (MAA) and NIP (MAA) were observed at 7 ppm of AME which were 94.32% and 52.69%, respectively. This indicated that 7 ppm of AME was the optimum concentration to obtain the highest adsorption efficiency of the synthesized MIP (MAA). The MIP (MAA) had much higher adsorption efficiency due to the availability of specific binding sites in its polymer matrix that are complementary to the template molecule (AME) in terms of size and shape. At 7 ppm, the AME adsorption by the MIP (MAA) reached its saturation or in other words, equilibrium has been achieved. At this point, the vacant binding sites in the MIP (MAA) cavity were filled up by the AME template molecule. This indicated that the adsorption process has reached its plateau and no more binding sites can be accessed by template molecules for further adsorption at much higher concentrations [68].

However, the adsorption efficiencies of both MIP (MAA) and NIP (MAA) were decreased gradually from 8 to 10 ppm because the synthesized polymers had reached their adsorption equilibrium at 7 ppm. After the adsorption equilibrium point, a further increase in AME initial concentrations will cause the accumulation of AME template molecule to gather around the MIP (MAA) binding sites which could hinder efficient adsorption of AME in the binding sites of MIP (MAA) cavity, hence decreases the adsorption efficiency. It can be further explained that the abundance of AME template molecules surrounding the MIP (MAA) will limit the binding ability between the AME template molecule and MIP (MAA) binding sites because each MIP (MAA) binding site can only attach with a single AME template molecule to increase the adsorption efficiency. Since the polymer dosages used were constant in all concentrations the number of available binding sites was also constant, however, the concentration of analyte was increased, explaining the increase in the number of moles of analyte in the solution which contributed to the decrease in MIP adsorption efficiencies [77].

In contrast, a similar trend was detected in the adsorption efficiencies of NIP (MAA) towards AME but in much lower efficiencies. This is because NIP (MAA) does not have specific binding sites in its cavity that can promote efficient adsorption with the AME template molecule since the non-specific binding sites were not corresponding to the AME template molecule [70].

### Effect of pH

Several pH conditions were applied to study their effect towards the adsorption efficiency of the synthesized polymers. The charges of both the analyte (template molecule) and the imprinted polymers are determined by the pH condition, thus affecting the electrostatic interactions (electrostatic attraction and repulsion) between the template monomer and imprinted polymer [78]. The surface charge is positively charged at a lower pH value but negatively charged at a higher pH value [68].

**Fig. 10:**
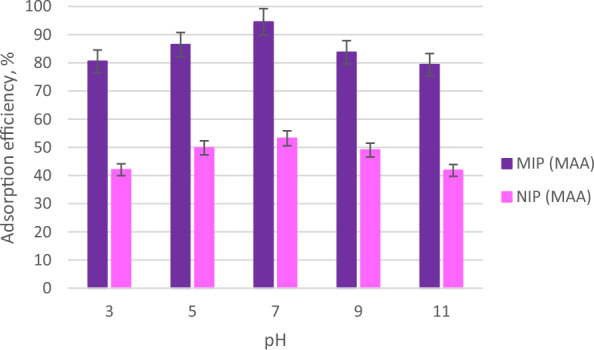
The adsorption efficiencies of MIP (MAA) and NIP (MAA) at several pH

Figure 10 represents the adsorption efficiencies of MIP (MAA) and NIP (MAA) at several pH (pH 3, pH 5, pH 7, pH 9 and pH 11) conditions. At pH 3 (strong acidic condition), the adsorption efficiencies of MIP (MAA) and NIP (MAA) were 80.56% and 42.06%, respectively. At pH 5 (weaker acidic condition), the adsorption efficiencies of MIP (MAA) and NIP (MAA) were 86.44% and 49.83%, respectively. A lower pH value of the analyte solution than the MIP will allow the MIP to be protonated and become positively charged, so it can simply adsorb the negatively charged analyte [79], increasing the adsorption efficiency. It was calculated that the highest adsorption efficiency of MIP (MAA) and NIP (MAA) was 94.51% and 53.21%, respectively at pH 7 (neutral condition) as shown in Fig. 10. Greater adsorption performance between the analyte and MIP can be achieved in the range of pH 5 to pH 7 because the analyte has practically no charge and the non-electrostatic interactions, for example, hydrogen bonding and π–π accumulation were involved in the adsorption process [80]. The highest adsorption of MIP at a certain pH value is governed by the neutral condition of the analytes and the hydrogen bonding interaction that occurs between the analyte and the MIP cavity [66].

Meanwhile, at pH 9 (weaker basic condition), the adsorption efficiencies of MIP (MAA) and NIP (MAA) were 83.71% and 49.06%, respectively. At pH 11 (strong basic condition), the adsorption efficiencies of MIP (MAA) and NIP (MAA) were 79.38% and 41.82%, respectively. At pH 9 and above, both the template molecule and the synthesized polymers were negatively charged which caused electrostatic repulsion interaction that decreased the interactions between the template molecule and the synthesized polymers, hence lowering the adsorption efficiency [81].

Hence, this explained that the synthesized MIP (MAA) was pH dependent as it required a neutral condition to obtain its highest adsorption efficiency. Overall, the synthesized MIP (MAA) presents a good imprinting effect and adsorption performance towards AME template molecule in several pH conditions as compared with NIP (MAA). The reason is MIP (MAA) contains specific binding sites in its polymer cavity while NIP contains non-specific binding sites in its polymer cavity for the adsorption of AME (template molecule).

### Effect of polymer dosage

**Fig. 11:**
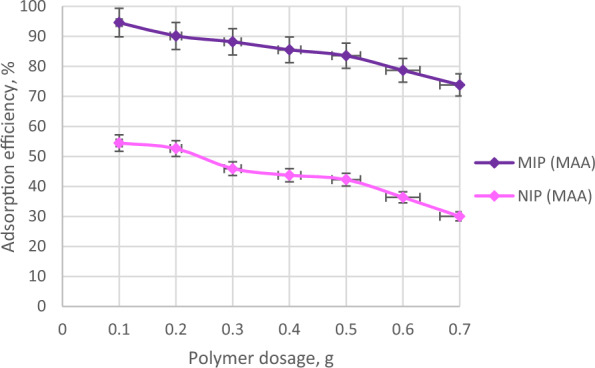
The adsorption efficiencies of MIP (MAA) and NIP (MAA) at various polymer dosages

Various polymer dosages were used to study its effect towards the adsorption efficiency of the synthesized polymers. Figure 11 describes the adsorption efficiencies of MIP (MAA) and NIP (MAA) at various polymer dosages such as 0.1 g, 0.2 g, 0.3 g, 0.4 g, 0.5 g, 0.6 g and 0.7 g. Commonly, the increase in polymer dosage will increase the number of active binding sites and surface area for the adsorption of the target analyte [82, 83]. In this study, it was observed that the increase in MIP (MAA) dosage leads to a gradual decrease in the adsorption efficiencies of polymer with AME as shown in Fig. 10. The higher polymer dosage did not contribute to higher adsorption efficiency of polymer with analyte as it can cause MIP aggregation [84] that hinders binding interactions between the analyte (template molecule) with the binding sites of the synthesized polymers [43]. Correspondingly, the NIP (MAA) had similar behaviour as MIP (MAA) but with much lower adsorption efficiencies. Based on Fig. 11, 0.1 g of MIP (MAA) and NIP (MAA) obtained the highest AME adsorption efficiencies of 94.58% and 54.44%, respectively. Meanwhile, this study was conducted until the dosage of 0.7 g of MIP (MAA) and NIP (MAA) which obtained the lowest adsorption efficiencies of 73.82% and 30.01%, respectively. Therefore, 0.1 g of MIP (MAA) is chosen as the optimum polymer dosage for the adsorption of 7 ppm AME solution.

### Kinetic studies

**Fig. 12:**
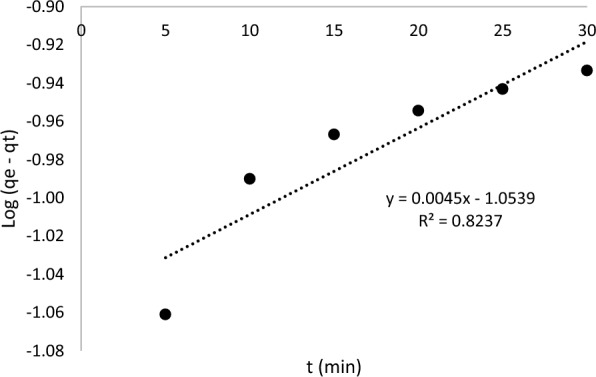
The pseudo-first-order kinetic model

**Fig. 13:**
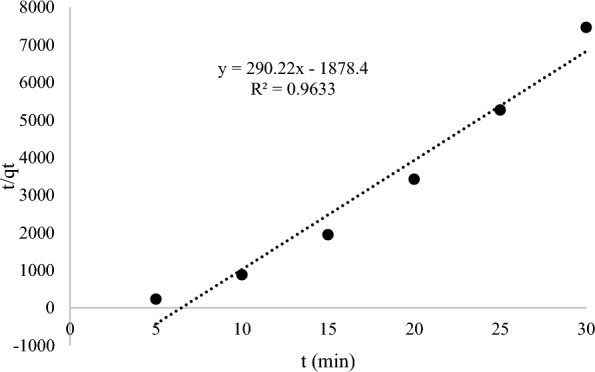
The pseudo-second-order kinetic model

**Fig. 14:**
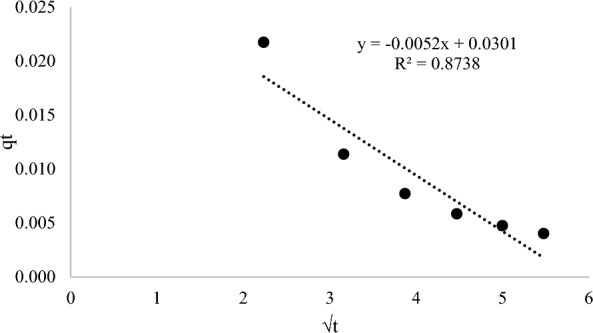
The intraparticle-diffusion kinetic model

Three different kinetic models such as pseudo-first-order, pseudo-second-order and intraparticle-diffusion were applied in kinetic studies to distinguish the adsorption process of AME on the surface of the MIP (MAA). The pseudo-first-order kinetic model assumes that the rate-limiting step is solute adsorption on the adsorbent’s surface, whereas the pseudo-second-order kinetic model assumes that the rate-limiting step is chemical sorption or chemisorption on the adsorbent’s surface. Furthermore, the intraparticle diffusion kinetic model depicts the adsorption process in which the rate of adsorption is determined by the rate at which the adsorbate diffuses towards the adsorbent. The kinetic results of AME adsorption onto the MIP (MAA) were evaluated by using different kinetic models including pseudo-first-order, pseudo-second-order and intraparticle-diffusion as depicted in Figs. 12, 13, and 14, respectively. The calculated values of the amount of AME (absorbate) adsorbed at equilibrium time (q_e_), rate constants (k) and correlation coefficients (R^2^) were given in Table 4. Among the three kinetic models, the pseudo-second-order kinetic model had the highest correlation coefficient (R^2^) value of 0.9633 and therefore it was selected as the best-fitted kinetic model for describing the AME (absorbate) uptake by the MIP (MAA) during the adsorption process. This explained that the chemical adsorption was primarily affecting the adsorption rates of absorbate [85]. Since the pseudo-second-kinetic model was the best-fitted kinetic model to describe the AME adsorption onto the MIP (MAA). Based on Table 4, the rate constants K_1_ and K_2_ derived from the pseudo-first-order and pseudo-second-order kinetic models have physical significance and implications for the adsorption mechanism. The correlation between K_1_ and K_2_ can shed light on the elements that influence the adsorption rate. Hence, the rate constant K_2_ (Table 4) from the pseudo-second-order kinetic model has physical significance since it measures the potential of AME template molecule reacting with MIP (MAA) binding sites. This is because a larger rate constant indicates that the reaction is more likely to take place, whereas a lower rate constant indicates that the reaction is less likely to take place.

**Table 4:** The kinetic models of MIP (MAA) of AME

Kinetic models									
Pseudo-1st-order			Pseudo-2nd-order			Intraparticle-diffusion			
q_e1_ (mg/g)	K_1_ (1/h)	R^2^	q_e2_ (mg/g)	K_2_ (g/mgh)	R^2^	C_o_ (mg/L)	K_dif_ (mg/h^1/2^ g)	C	R^2^
0.3486	**–** 0.0045	0.8237	0.0034	44.840	0.9633	1.3519	− 0.0052	0.0301	0.8738

### Adsorption isotherms

**Fig. 15:**
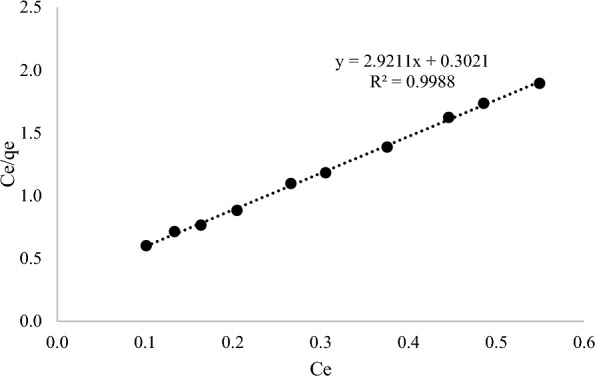
The Langmuir isotherm model

**Fig. 16:**
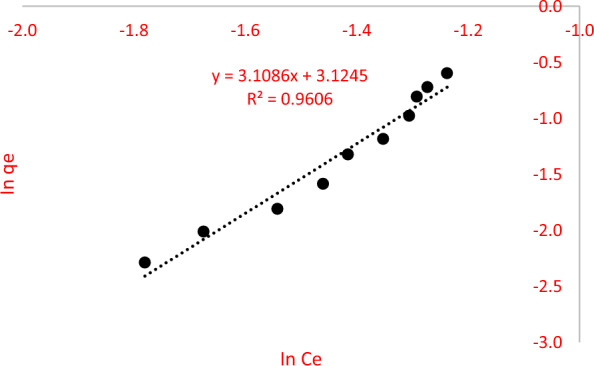
The Freundlich isotherm model

Generally, the Langmuir adsorption isotherm is defined as mono-layer adsorption of AME by MIP (MAA) while the Freundlich adsorption isotherm is defined as multi-layer adsorption of AME by MIP (MAA). The adsorption isotherm study of MIP (MAA) was analysed based on the Langmuir and Freundlich isotherm models as shown in Figs. 15 and 16, respectively. Based on Figs. 15 and 16, C_e_ refers to the AME concentration at equilibrium, meanwhile q_e_ refers to the amount of AME adsorbed at equilibrium time. Moreover, the values of Langmuir constants (q_max_, K_L_ and R_L_) and their correlation coefficient (R^2^), as well as the Freundlich constants (K_F_, *n* and 1/*n*) and their correlation coefficient (R^2^) were displayed in Table 5. It showed that the Langmuir isotherm model of AME-MIP (MAA) had a higher correlation coefficient (0.9988) than the Freundlich isotherm model (0.9606). Hence, the Langmuir isotherm model is the best to describe the chemical adsorption between AME that took place on the surface of the MIP (MAA). The Langmuir isotherm model indicates that the adsorption of absorbate was monolayer and the adsorption sites of the absorbent were homogenous [86].

**Table 5:** The adsorption isotherm models of MIP (MAA) of AME

Langmuir constants				Freundlich constants			
q_max_ (mg/g)	K_L_ (L/mg)	R_L_	R^2^	K_F_ (mg/g)	*n*	1/*n*	R^2^
0.3423	9.6693	0.0803	0.9988	22.75	0.3217	3.1086	0.9606

### Selectivity of MIP

The selectivity test was employed to investigate the sensing property of synthesized MIP (MAA) towards AME. In this study, Cyanazine was selected as the competitive compound the structural analogue of AME. The selectivity of MIP (MAA) towards AME (template compound) and Cyanazine (competitive compound) was analysed by using UV–Vis spectrophotometer. Then, the selectivity of MIP (MAA) was calculated based on the distribution ratio (K_D_), selectivity coefficients (K_sel_) and relative selectivity coefficient (k′) as shown in Table 6. Initially, the distribution ratio (K_D_) of both MIP (MAA) and NIP (MAA) for their selective adsorption towards AME and Cyanazine was calculated. It showed that the distribution ratio (K_D_) of AME by MIP (MAA) is much higher than the distribution ratio of Cyanazine, its competitive compound because MIP (MAA) has specific binding sites that are complementary with the AME template molecule. However, the distribution ratio (K_D_) of AME by NIP (MAA) is lower than the distribution ratio of Cyanazine due to the non-specific binding sites in the NIP matrix.

**Table 6:** The distribution ratio, selectivity coefficients and relative selectivity coefficient of MIP (MAA) and NIP (MAA)

	K_D_ MIP (MAA)	K_D_ NIP (MAA)	K_sel_	k′
Ametryn (template molecule)	94.75	31.51	2.52	–
Cyanazine (competitive compound)	37.67	33.29	0.95	2.66

Hence, the selectivity coefficient of AME was greater than that of Cyanazine, explaining that the effect of the imprinting method of MIP (MAA) and its selectivity towards AME was remarkably successful. This is due to the synthesized MIP containing binding sites corresponding to its template molecule as compared to the competitive compound [57]. The relative selectivity coefficient was 2.66, indicating that MIP (MAA) has been good selectivity towards its compound of interest (AME). The value of the relative selectivity coefficient greater than 1 indicated that the synthesized MIP showed a good performance as molecular recognition [83]. Therefore, the synthesized MIP (MAA) has selectively recognized the AME template molecule because the synthesized MIP (MAA) had specific binding sites that are complementary to the AME template molecule.

### Removal of AME from environmental samples

The application of the synthesized MIP (MAA) was performed on spiked samples including distilled water (Environmental Lab, UNIMAS), tap water (Environmental Lab, UNIMAS) and river water (Samarahan River) to distinguish the removal efficiency of AME.

A successful application of synthesized MIP for the removal of AME can be conducted on real samples especially environmental samples (tap water and river water). Each of these samples was initially spiked with AME to determine the removal efficiency of synthesized MIP (MAA) as the MIP (MAA) has specific binding sites that can specifically bind with AME.

Based on Table 7, the removal efficiency of AME from distilled water, tap water and river water by using MIP (MAA) was 95.01%, 90.24% and 88.37%, respectively. Meanwhile, the removal efficiency of AME from distilled water, tap water and river water by using NIP (MAA) were 54.97%, 51.31% and 49.45%, respectively. Hence, MIP (MAA) had an outstanding percentage of AME removal from different water samples than NIP (MAA) due to the presence of specific binding sites in MIP (MAA) that are highly accessible for AME template molecule, resulting in a greater imprinting effect.

**Table 7:** Removal of AME from environmental samples

Samples	Amount of CYZ added (µg/mL)	Amount of CYZ found (µg/mL)	MIP (MAA) recovery (%)	Standard deviation, SD	Relative standard deviation, RSD	Amount of CYZ found (µg/mL)	NIP (MAA) recovery (%)	Standard deviation, SD	Relative standard deviation, RSD
DIW	25	23.75	95.01	0.0097	0.01	13.74	54.97	0.0187	0.03
Tap water	25	22.56	90.24	0.0379	0.04	12.83	51.31	0.0563	0.11
River water	25	22.09	88.37	0.0045	0.01	12.36	49.45	0.0050	0.01

### Reusability of MIP (MAA) in environmental samples

The reusability experiments of the synthesized MIP (MAA) were conducted by using environmental (DIW, tap water and river water) to determine its reusability characteristic for the rebinding of AME several times with noticeably altering its molecular recognition efficiency. Table 8 indicated the effect of the reusability of MIP (MAA) for the removal of AME in environmental samples. Based on Table 8, the removal efficiency of AME in DIW, tap water and river water were decreased gradually from the first cycle to the third cycle. This showed that the MIP (MAA) has good reusability characteristic because it can retain its high rebinding efficiency and selectivity upon multiple times of application.

**Table 8:** The reusability of MIP (MAA) for the removal of AME

Sample	Removal efficiency of AME by using MIP (MAA), %		
	DIW	Tap water	River water
Adsorption–desorption cycle			
Cycle-1	95.03	90.27	88.37
Cycle-2	93.74	86.56	83.06
Cycle-3	90.40	83.57	80.01

## Conclusions

The synthesis of MIPs of AME via precipitation polymerization has successfully produced imprinted polymers for efficient and selective removal of AME from contaminated water samples. Among the three synthesized polymers, MIP (MAA) was selected as the best imprinted polymer due to its highest batch binding efficiency. Besides that, the generated MIP (MAA) was a micro-spherical polymer (0.52 µm) with good porosity (103.1369 Å) that promotes better binding properties. It was deduced that MIP (MAA) had an outstanding removal efficiency of AME at optimum conditions of 6 ppm of AME concentration, solution of pH 7, 0.1 g of polymer dosage and 210 min of contact time. The synthesized MIP (MAA) was more selective towards AME (template molecule) than Cyanazine (competitive compound) with a relative selectivity coefficient of 2.66. The MIP (MAA) was successfully applied for the removal of AME in distilled water, tap water and river water with a good efficiency of 95.01%, 90.24% and 88.37%, respectively.

## Data Availability

All data generated or analysed during this study are included in this article.

## Abbreviations

AME: Ametryn
MIP: Molecular imprinted polymer
NIP: Non-imprinted polymer
MAA: Methacrylic acid
AAm: Acrylamide
4VP: 4-Vinyl pyridine
EGDMA: Ethylene glycol dimethacrylate
AIBN: Azo-bis-isobutyronitrile
FTIR: Fourier transform infrared spectroscopy
FESEM: Field emission scanning electron microscopy
EDX: Energy dispersive X-ray
TGA: Thermogravimetric analysis
BET: Brunauer–Emmett–Teller
UV–Vis: Ultra violet–visible spectrophotometry
RP-HPLC: Reversed-phase high performance liquid chromatography
